# The potential of *Trichoderma asperellum* for degrading wheat straw and its key genes in lignocellulose degradation

**DOI:** 10.3389/fmicb.2025.1550495

**Published:** 2025-04-23

**Authors:** Qijun Zhu, Weiwei Liu, Liye Song, Zhenzhen Guo, Zhiyao Bian, Yunsheng Han, Hongying Cai, Peilong Yang, Kun Meng

**Affiliations:** ^1^Institute of Feed Research, Chinese Academy of Agricultural Sciences, Beijing, China; ^2^College of Animal Science and Technology, Hebei Agricultural University, Baoding, China

**Keywords:** wheat straw, lignocellulose degradation, enzymatic activity, transcriptome, reducing sugars, *Trichoderma asperellum*

## Abstract

This study explored *Trichoderma asperellum*’s lignocellulose degradation potential in wheat straw (WS) and NaOH-treated WS via solid-state fermentation (SSF) over 30 days. Compared to the control, WS treated with *T. asperellum* (TW) and NaOH-treated WS with *T. asperellum* (TN) showed increased dry matter loss rates of 15.67 and 15.76%, respectively. Cellulose degradation reached 33.51 and 28.00%, while hemicellulose degradation increased to 31.56 and 63.86%. Crude protein (CP) content rose to 10.96 and 7.44%, and reducing sugar content to 10.86 and 12.41 mg/g, respectively. *T. asperellum* effectively reduced lignocellulose content and enhanced substrate nutrition, supporting subsequent uses of WS as fertilizer, feed, or for bioethanol production. Enzymatic activity and structural analyses were performed to further confirm the lignocellulose-degrading ability of *T. asperellum* and to analyze the degradation mechanisms. Transcriptomic analysis revealed that, compared with the control group, the TN group had 4,548, 4,399, and 6,051 differentially expressed genes (DEGs) at 5, 10, and 30 days, respectively, mainly involved in cellulose and hemicellulose degradation, carbohydrate metabolism, carbohydrate transport, glycoside hydrolases, and polysaccharide binding. *T. asperellum* can modify lignin by expressing dye-decolorizing peroxidase genes, and multiple key genes were identified for further research into its genetic regulation in lignocellulose degradation.

## 1 Introduction

In China, the annual production of crop residues has remained at approximately 970 million tons from 2014 to 2021, with wheat straw (WS) being the most abundant agricultural byproduct after corn (Sun et al., 2021). Wheat cultivation is predominantly concentrated in North China, East China, and Northwest China, which contributes to the substantial generation of WS and consequently poses challenges in its management and utilization (Zhao et al., 2017). As an agricultural byproduct with considerable energy storage, it primarily contains a large amount of high-energy substances such as cellulose and hemicellulose, as well as substances like lignin, pectin, and inorganic salts (Reyes et al., 2021). Despite its high utilization value, open-field straw burning still persists, which not only wastes valuable resources but also causes serious environmental pollution (Lou et al., 2022). The use channels and fields of WS are varied in their promotion of energy saving, emission reduction, and waste-to-resource transformation. They include industrial uses like papermaking, bioethanol production, and biohydrogen generation, as well as agricultural uses like fertilizerization and feedization. These diversified utilization approaches provide new insights and directions for the sustainable development of WS. However, the tightly compact and recalcitrant structure of lignocellulose hinders the efficiency of these conversion processes (Wu et al., 2022). The primary goal is to reduce the content of lignocellulose to a utilizable range and to repurpose and process by-products.

Currently, some methods are used to break down complex lignin and improve the accessibility of microbial enzymes to convert cellulose and hemicellulose into monosaccharides (Mosier et al., 2005; Galbe and Wallberg, 2019). They can generally be divided into physical, chemical, physicochemical, and biological methods (Becker and Wittmann, 2019). One of the most common and efficient way to treat lignocellulose is through the synergistic treatment of chemical and biological methods (Mohanram et al., 2015). Chemical treatment aims to disrupt the structure of lignocellulose with chemical reagents in a short period, thereby accelerating its degradation by microorganisms (da Costa Sousa et al., 2009; Wu et al., 2022). Biological treatment mainly changes the content and structure of lignocellulose by secreting various degradation enzymes and metabolic products (Hou et al., 2020), which is environmentally friendly but has a prolonged degradation period.

The solid-state fermentation (SSF) of WS mainly involves the process where microorganisms degrade and utilize the lignocellulose and metabolize it to produce usable nutrients. WS has a naturally low organic digestibility energy. However, SSF boosts protein and sugar content of WS, enriching its nutritional content and improving palatability. Nutritional components primarily refer to protein, reducing sugars, polysaccharides, fat, and other nutrients after SSF (Zhang Y. et al., 2022). Currently, research on the fungal degradation of lignocellulose is more prevalent, compared to bacteria, which produce extracellular enzymes that are not only diverse and complex but also more efficient. Among them, white-rot fungi, brown-rot fungi and soft-rot fungi are representatives of efficient fungi that degrade lignocellulose in nature. Soft rot fungi are predominantly fungi from the Ascomycetes and Deuteromycetes that are capable of degrading lignocellulose, including genera such as *Trichoderma*, *Aspergillus* and *Rhizopus*, etc. (Sista Kameshwar and Qin, 2018). Soft rot fungi are characterized by a broader degradation and utilization of cellulose and hemicellulose, and also demonstrate some effectiveness in lignin degradation (Goodell et al., 2020). Among them, *Trichoderma reesei* is one of the most extensively studied for cellulase degradation, with its ability to secrete large amounts of cellulases and hemicellulases making it a primary microorganism for enzyme preparation (Havukainen et al., 2021). Additionally, *Trichoderma harzianum* and *Trichoderma viride* exhibit similar characteristics (Iqtedar et al., 2015; Zhang Y. et al., 2020). In our preliminary experiments, we discovered a strain of fungi from the *Trichoderma* genus, *Trichoderma asperellum*, which showed significant potential in the degradation of lignocellulose. However, the mechanisms and pathways of lignocellulose degradation by soft-rot fungi remain poorly understood (Woiciechowski et al., 2013).

As global attention to sustainable development intensifies, developing efficient biomass conversion technologies becomes crucial. The potential of *Trichoderma* as a biological catalyst in lignocellulose degradation could revolutionize bioenergy and bio based chemical production (Lynd et al., 2002)*. T. asperellum* has been extensively documented for its role as a biocontrol agent in enhancing the disease resistance of crops (Jiang et al., 2016; Tao et al., 2020; Yadav et al., 2021), its application as a soft rot fungus in the fermentation of WS has been less extensively reported in the literature. In particular, our understanding of the situation expression of its genes encoding lignocellulose-degrading enzymes remains very limited. This study designed the degradation of lignocellulose in WS treated with two different methods by *T. asperellum*, attempting to explore the following: (1) the degradation efficiency of *T. asperellum* on different lignocellulosic substrates; (2) the mechanisms of lignocellulose degradation by *T. asperellum* and the key genes involved in this process, which are central to understanding its biodegradative capabilities. By exploring its degradation mechanisms, this study provides a scientific basis for sustainable biomass transformation.

## 2 Materials and methods

### 2.1 Preparation of raw materials

WS was purchased from the scientific procurement platform of the Chinese Academy of Agricultural Sciences. A hammer mill was used to pulverize the wheat straw, and then a sieve was employed to filter out WS powder with particle sizes of 20–40 mesh. The powder was placed in an oven at 65°C and dried for 2 days. After drying, the WS powder was stored in self-sealing bags for later use. NaOH-pretreated WS was prepared as follows: WS powder of size 20–40 mesh was soaked in a 3% NaOH (w/v) solution with a solid–liquid ratio of 1:10 for 24 h at room temperature, and then rinsed with distilled water until the pH reached neutrality, placed in an oven at 65°C until a constant weight was achieved. NaOH-pretreated WS was also stored in self-sealing bags for later use.

### 2.2 Inoculation of strains and SSF

The strain of *T. asperellum* (CGMCC 41476) was isolated from naturally fermented WS in the laboratory and preserved in the Institute of Microbiology, Chinese Academy of Sciences. Potato Dextrose Broth (PDB) was used for the enrichment culture of *T. asperellum* and Potato Dextrose Agar (PDA) was used for its growth on plates. The mycelial pellets are obtained by transferring a quarter of a PDA agar plate of well-grown *T. asperellum* into PDB medium and cultivating on a shaking bed at 30°C and 200 rpm for 5 days. The mycelium amount, equivalent to half the volume of the sieve, was filtered out using a 40-mesh handheld cylindrical sieve with a diameter of 5 cm and a height of 2 cm for subsequent fermentation. Respectively, 10 g of untreated WS and 10 g of WS pretreated with 3% NaOH were placed into glass petri dishes (12 cm in diameter), and then sterilized at 115°C for 20 min. The substrates (10 g) and the nutrient solution were mixed at a mass ratio of 1:2, adding mycelium pellets and mixing evenly. The nutrient solution composition of SSF is glucose 0.025 g/mL, ammonium sulfate 0.015 g/mL, dipotassium phosphate and magnesium sulfate, each 0.0025 g/mL. SSF was carried out in a 30°C incubator. The control group (0d) involved the SSF substrates undergoing processing without the inoculation of microorganisms. After inoculation, samples were taken on the 5th, 10th, 20th, and 30th days to measure the content of cellulose, hemicellulose, lignin, crude protein (CP) and reducing sugars after fermentation. At the same time, 2 g of fresh sample were taken every 5 days after the start of SSF to measure enzyme activity. Three parallel trials were performed for each group to get the average value.

### 2.3 Preparation and collection of crude enzyme solutions

2.0 g of fresh sample from the SSF was added it to 12 mL of acetic acid/sodium acetate buffer solution (pH 5.0, 10 mM/L), and incubated on a shaking bed at 30°C and 200 rpm for 2 h. Then, it was centrifuged at 4°C and 6,000 rpm for 5 min, after which the supernatant was collected and divided into 1.5 mL centrifuge tubes for storage at −20°C for determination of lignocellulolytic enzyme activities (Lu et al., 2023).

### 2.4 Determination of lignocellulose content and its degradation rate

The content of crude fiber (CF), neutral detergent fiber (NDF), acid detergent fiber (ADF), and acid-insoluble lignin (ADL) was determined using the Van Soest washing fiber analysis method (Van Soest et al., 1991). The content of cellulose, hemicellulose, and lignin is calculated as follows: Cellulose (%) = ADF - ADL; Hemicellulose (%) = NDF - ADF; Lignin (%) = ADL. The degradation rate can be calculated using the following formula: degradation rate ( $\%)=1-\frac{m_{2}*x_{2}}{m_{1}*x_{1}}$ *100%.

m_1_ (g): the mass of dry matter before SSF.

m_2_ (g): the mass of dry matter after SSF.

x_1_ (%): the content of lignocellulose before SSF.

x_2_ (%): the content of lignocellulose after SSF.

### 2.5 Determination of nutrient content

#### 2.5.1 Determination of crude protein content

The Dumas nitrogen analyzer was used to determine the CP content of the WS (Saint-Denis and Goupy, 2004).

#### 2.5.2 Determination of reducing sugars content and monosaccharides

According to Miller’s description (Miller, 1959), the 3,5-dinitrosalicylic acid (DNS) method was used to quantify reducing sugars derived from fermented WS. The qualitative and semi-quantitative analysis of monosaccharides derived from fermented WS was carried out using HPLC (Shimadzu LC-20 AD, Japan). Chromatographic conditions: Xtimate C18 column, with a mobile phase consisting of a mixture of 0.05 M potassium dihydrogen phosphate solution (pH 6.70) and acetonitrile in an 83:17 ratio, a flow rate of 1.0 mL/min, column temperature of 30°C, detection wavelength of 250 nm, and an injection volume of 20 μL.

### 2.6 Determination of lignocellulolytic enzyme activities

#### 2.6.1 For cellulase enzymes

Filter paper activity (FPase) was determined by mixing 50 mg of Whatman No. 1 filter paper (0.2 × 1.2 cm) with 0.2 mL of enzyme solution and 0.5 mL of acetic acid–sodium acetate buffer (50 mM, pH 4.8) in a water bath at 50°C for 30 min. Then, 0.8 mL of DNS solution was added to terminate the enzyme reaction, followed by boiling for 5 min. The absorbance of the mixture was measured at 540 nm. Carboxymethylcellulase (CMCase) activity was measured by mixing 0.2 mL of crude enzyme solution with 0.2 mL of 1% carboxymethyl cellulose sodium buffer solution. The mixture was incubated at 50°C for 30 min, then 0.6 mL of DNS solution was added to terminate the enzyme reaction, followed by boiling for 10 min. After cooling to room temperature, the absorbance was measured at 540 nm (Berghem et al., 1976). Exo-β-1,4-glucanase activity was determined by mixing 0.1 mL of crude enzyme with 0.4 mL of p-nitrophenyl cellobioside (pNPC) at 37°C for 60 min (Ravindran and Jaiswal, 2016). After adding 1 mL of 1 M Na_2_CO_3_ solution to terminate the reaction and mixing evenly, the absorbance was measured at 400 nm. The β-glucosidase activity was measured by mixing 0.1 mL of crude enzyme with 0.4 mL of p-nitrophenyl-β-D-glucopyranoside (pNPG, 5 mM) at 37°C for 30 min. Then, 1 mL of 1 M Na_2_CO_3_ solution was added to terminate the reaction and mixed evenly. The absorbance was measured at 400 nm (Zhang et al., 2021).

#### 2.6.2 For hemicellulase enzymes

Xylanase activity was measured by mixing 0.2 mL of crude enzyme with 0.2 mL of xylan (1%) in 0.3 mL of citrate–phosphate buffer solution (50 mM, pH 5.0) at 50°C for 30 min then adding 0.3 mL of DNS solution to terminate the enzyme reaction, followed by boiling for 5 min (Cho et al., 2020). The absorbance of the mixture was measured at 540 nm (Miller, 1959). Beta-xylosidase activity was determined by mixing 0.2 mL of crude enzyme solution with 0.05 mL of p-nitrophenyl-β-D-xylopyranoside in 0.35 mL of 50 mM Tris–HCl buffer (pH 7.0) and incubating at 45°C for 20 min. The reaction was terminated with 0.4 mL of 1 M Na_2_CO_3._ After allowing the color to develop at room temperature for 5 min, the absorbance of the product at 400 nm was recorded (Liu et al., 2019).

#### 2.6.3 For lignin enzymes

Laccase (Lac) activity was determined by mixing 0.15 mL of enzyme solution with 0.85 mL of 10 mmol/L ABTS (Edens et al., 1999). The absorbance was measured at 420 nm after mixing for 10 s, then the mixture was incubated in a 45°C water bath for 3 min, and the absorbance at 420 nm was measured again. MnP activity was determined by mixing 0.08 mL of enzyme solution, 0.04 mL of 0.4 mmol/L MnSO4, 0.08 mL of dimethoxyphenol, 0.02 mL of 0.4 mmol/L H_2_O_2_ solution, and 0.58 mL of 0.2 mol/L malic acid buffer at pH 4.5. After mixing, the absorbance at 470 nm was measured at the 10th second, and then another measurement was taken after the reaction had proceeded for 10 min. Lignin peroxidase activity (LiP) was determined by the oxidation of veratryl alcohol (VA) to veratraldehyde (Tien and Kirk, 1988). The reaction mixture consisted of 1 mL, 0.1 mL of enzyme solution, 0.2 mL of VA, 0.6 mL of sodium tartrate buffer (pH 3.0, 50 mmol/L), and 0.1 mL of 0.4 mmol/L H_2_O_2_ to initiate the reaction. The absorbance was measured at 310 nm after mixing for 10 s, and then another reading was taken after 5 min of reaction.

An enzyme activity unit (U/Kg) was defined as the amount of enzyme that consumes 1 μmol of glucose from the substrate per minute for CMCase and FPase; or 1 μmol of reducing sugars produced from the substrate per minute for xylanase; or 1 μmol of p-nitrophenol from the substrate per minute for β-glucosidase, exo-β-1,4-glucanase and β-xylosidase; or 1 μmol of ABTS, DMP and VA oxidized per minute for Lac, MnP, and LiP, respectively.

### 2.7 Scanning electron microscopy analysis

The samples were lyophilized using a freeze-dryer for 2 days. Following this, the samples were subjected to metal sputtering using a gold-coating apparatus to enhance conductivity and imaging quality. The coated samples were then examined under a Zeiss Sigma 300 scanning electron microscope (Germany). Observations were made under high-vacuum conditions at various magnifications to scrutinize the surface morphology of the samples.

### 2.8 Fourier transform infrared spectroscopy analysis

The lignocellulosic materials from various fermentation periods were dried to a constant weight at 65°C. Fourier transform infrared spectroscopy (FTIR) was utilized to identify the primary organic functional groups present in the lignocellulose. A fourier transform infrared spectrophotometer (Thermo-Nicolet 470, United States) was employed to acquire the FTIR absorption spectra in the spectral range of 400–4,000 cm^−1^, with a scanning speed of 8 cm/s and 64 scans accumulated for each analysis. The origin was used to plot the sample spectra.

### 2.9 Transcriptome sequencing and analysis

#### 2.9.1 Cultivation conditions

*T. asperellum* grown on NaOH-pretreated WS, cultivated for the 5th, 10th, and 30th days in a 30°C incubator, were collected and then frozen in liquid nitrogen and stored at −80°C for subsequent transcriptomic analysis. *T. asperellum* was cultivated on PDA at 30°C for 5 days, and the mycelium obtained was used as the control group (CK).

#### 2.9.2 Transcriptome analysis

Total RNA was extracted from the samples using the QIAzol Lysis Reagent kit (Qiagen, Germany) and treated with DNase I. The concentration and purity of the extracted RNA were measured using a NanoDrop 2000 spectrophotometer (Thermo Fisher Scientific), and the integrity of the RNA was assessed via agarose gel electrophoresis. The RNA integrity number was determined using an Agilent 5300. PolyA-structured mRNA was enriched using oligo (DT) magnetic beads and then randomly fragmented using a fragmentation buffer. These fragments were reverse-transcribed into the first-strand cDNA using random primers and reverse transcriptase. Subsequently, the second-strand cDNA was synthesized using dNTPs and DNA polymerase I, forming a stable double-stranded structure. The cDNA library was constructed using the Illumina^®^ Stranded mRNA Prep, Ligation (Illumina, San Diego, CA). The library was sequenced on the Illumina NovaSeq 6000 platform by Shanghai Meiji Biomedical Technology Co., Ltd. (Beijing, China). Adapter sequences, low-quality reads, N (representing uncertain base information), and sequences that were too short were removed from the raw data to enhance the quality of the sequencing data (Bolger et al., 2014). The quality-controlled raw data were aligned to the reference genome and assessed for alignment quality using the HiSat2 software. The expression levels of genes and transcripts were quantified using the software RSEM to facilitate subsequent analysis of differential gene/transcript expression among different samples, and to reveal gene regulatory mechanisms by integrating sequence functional information (Roberts et al., 2011). The DESeq2 software was used for differential expression gene (DEG) analysis (Benevenuto et al., 2019). DEGs were identified using thresholds of FDR and |log2FC| ≥ 1. Gene Ontology (GO) and Kyoto Encyclopedia of Genes and Genomes (KEGG) pathway enrichment analysis of DEGs was performed using R software based on the hypergeometric distribution. Furthermore, functional enrichment analysis, including GO and KEGG, was conducted to determine which DEGs were significantly enriched in GO terms and metabolic pathways compared to the whole transcriptome background, with a Bonferroni-corrected *p*-value ≤ 0.05. Goatools and KOBAS were used for the analysis of GO functional enrichment and KEGG pathway analysis, respectively. Heatmaps were generated using row normalization based on z-score standardization. The complete transcriptomic information related to this study has been deposited under the accession number PRJNA1173924 at the National Center for Biotechnology Information (NCBI).

#### 2.9.3 Real-time quantitative PCR assays

Real-time quantitative PCR (RT-qPCR) assays were conducted to quantify the expression of four lignocellulose-related DEGs. Total RNA was extracted using the modified CTAB method, followed by reverse transcription into cDNA using the SweScript All-in-One RT SuperMix for qPCR kit (Vazyme, China). A 2.5 μL aliquot of cDNA template was added to a 20 μL reaction mixture in the SuperReal PreMix Plus kit (TIANGEN, China), employing a two-step PCR protocol. Quantitative PCR was performed on the C1000 TouchTM CFX Real-Time PCR Detection System (Bio-Rad Laboratories, United States). A target gene expression was normalized to the housekeeping gene (α-tubulin) expression and compared among samples using the 2^−ΔΔCt^ method. Primer sequences are listed in Supplementary Table S1.

### 2.10 Data analysis

The experiments were conducted in triplicate, and the average values were calculated. Statistical analysis was performed using SPSS 24.0. Subsequently, the relevant data were initially assessed for normality and homogeneity of variance. Upon confirming that the data satisfied the prerequisites for these tests, a one-way analysis of variance (ANOVA) was performed. This was succeeded by LSD post-hoc tests and Duncan’s multiple range test to identify significant differences, which were indicated by distinct letters. Different letters were used to denote significant differences (*p* < 0.05).

## 3 Results

### 3.1 Chemical composition of lignocellulose and determination of degradation rate

**Figure 1:**
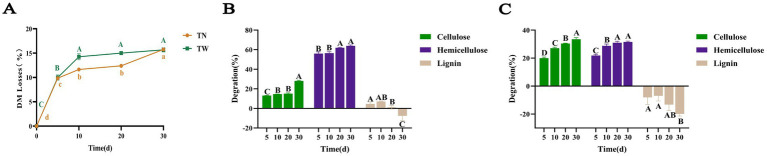
The loss of dry matter **(A)** and the degradation rate of lignocellulose in SSF of TN **(B)** and TW **(C)**. Different letters indicate significant differences between treatments (*p* < 0.05).

*T. asperellum* was used to perform SSF on both WS and NaOH-pretreated WS. After SSF with *T. asperellum*, the contents of CF, cellulose, hemicellulose, and lignin in the TN and TW groups changed significantly, with specific data shown in Tables 1, 2, respectively. During the dynamic fermentation process, both the TN and the TW groups exhibited a decrease in the content of CF, cellulose, and hemicellulose to varying degrees, with further reductions as the fermentation time progressed. Compared with 0d, the TN group showed reductions in the contents of CF, cellulose, and hemicellulose to 58.1, 54.49, and 6.77% on day 30, respectively. In the TW group, these contents further decreased to 34.22, 29.85, and 23% on day 30, respectively. The dry matter loss rate during the SSF by *T. asperellum* was shown in Figure 1A. It was found that the TN and TW groups had a faster rate of dry matter loss from day 0 to day 10, after which the rate slowed but still showed an upward trend, especially in the TN group, which showed a more significant increase from day 20 to day 30. On day 30, the dry matter loss rates for the TN and TW groups were 15.76 and 15.67%, respectively. It reflected the lignocellulose degradation rates of the TN and TW groups (Figures 1B,C), revealing that the cellulose degradation rate in the TN group was generally lower than that in the TW group, while the hemicellulose and lignin degradation rates were generally higher in the TN group than in the TW group. Similarly, the degradation rates of cellulose and hemicellulose in both the TN and TW groups gradually increased with fermentation. On day 30, the cellulose and hemicellulose degradation rates in the TN group were 28.00 and 63.86%, respectively, and in the TW group, they were 33.51 and 31.56%, respectively. Thus, the aforementioned tests demonstrated that *T. asperellum* is capable of efficiently breaking down lignocellulose in WS. *T. asperellum* exhibits a higher lignocellulose degradation efficiency in the TW group, according to a number of degradation indices.

**Table 1:** The content of CF and lignocellulose of TN varies with fermentation time.

Time (d)	CF (%)	Cellulose (%)	Hemicellulose (%)	Lignin (%)
0	64.87 ± 0.04^a^	63.78 ± 0.15^a^	15.78 ± 0.33^a^	7.14 ± 0.15^c^
5	64.84 ± 0.55^a^	61.44 ± 0.06^b^	7.75 ± 0.25^b^	7.55 ± 0.16^c^
10	64.04 ± 0.22^b^	61.62 ± 0.16^b^	7.77 ± 0.25^b^	7.51 ± 0.03^c^
20	63.67 ± 0.14^b^	61.78 ± 0.52^b^	6.85 ± 0.10^c^	8.05 ± 0.15^b^
30	58.10 ± 0.31^c^	54.49 ± 0.58^c^	6.77 ± 0.18^c^	9.11 ± 0.41^a^

The data represent the average of three measurements, with the numbers being the mean values ± standard deviation of the three replicates, and lowercase letters following the data in the same column indicate significant differences (*p* < 0.05).

**Table 2:** The content of CF and lignocellulose of TW varies with fermentation time.

Time (d)	CF (%)	Cellulose (%)	Hemicellulose (%)	Lignin (%)
0	39.32 ± 0.14^a^	37.95 ± 0.64^a^	28.42 ± 0.56^a^	6.89 ± 0.16^d^
5	37.30 ± 0.29^b^	33.76 ± 0.33^b^	24.69 ± 0.28^b^	8.28 ± 0.39^c^
10	36.14 ± 0.83^c^	32.26 ± 0.32^c^	23.59 ± 0.41^c^	8.59 ± 0.29^c^
20	35.40 ± 0.54^d^	31.03 ± 0.90^d^	23.09 ± 0.17^c^	9.17 ± 0.34^b^
30	34.22 ± 0.31^e^	29.85 ± 0.48^e^	23.00 ± 0.19^c^	9.77 ± 0.13^a^

The data represents the average of three measurements, with the numbers being the mean values ± standard deviation of the three replicates, and lowercase letters following the data in the same column indicate significant differences (*p* < 0.05).

### 3.2 Determination of nutrient content in SSF

**Figure 2:**
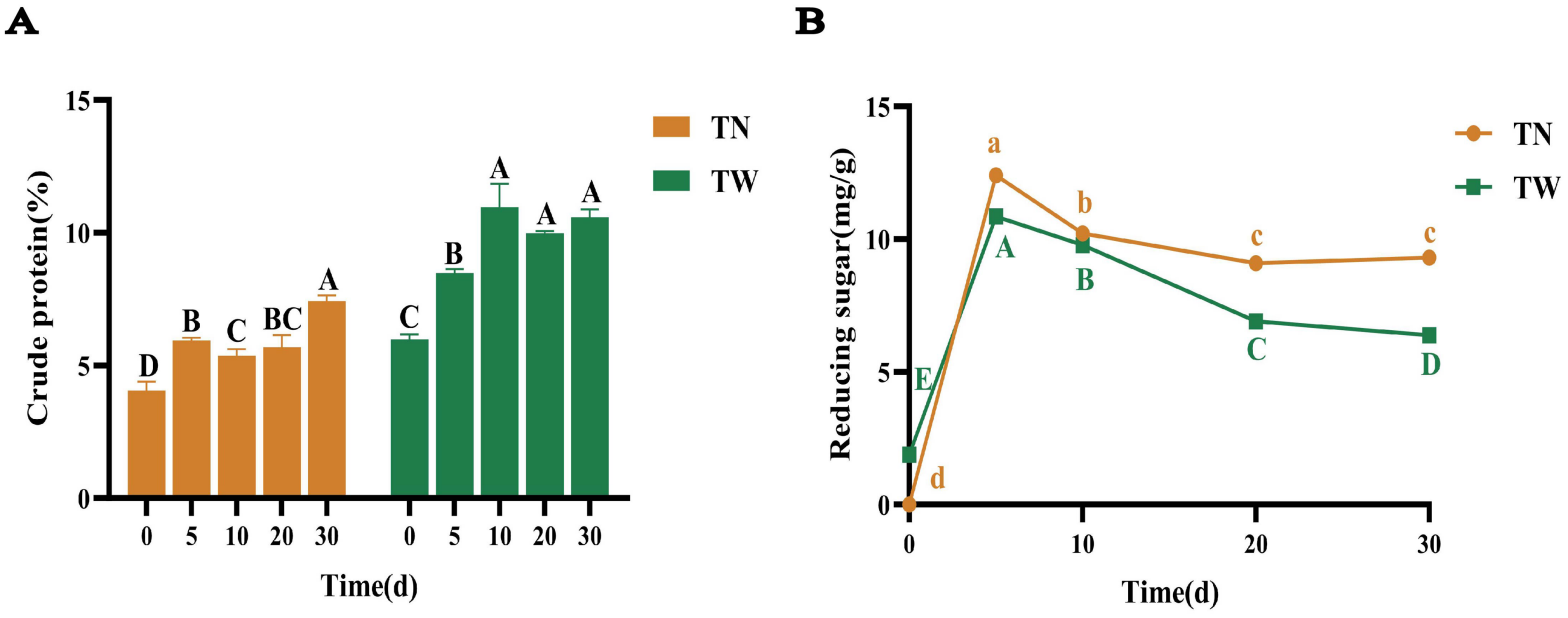
The CP **(A)** and reducing sugars **(B)** content in SSF of TN and TW groups. Different letters indicate significant differences at a *p*-value of < 0.05.

As fermentation proceeded, the CP content reached 7.44% on day 30 in the TN group, which was significantly increased to 1.84 times that of the day 0; there was no significant increase in CP content among the 10th, 20th, and 30th days in TW group, but compared to day 0, the CP content was 10.96% on the 10th day, which was significantly increased to 1.83 times that of the original (Figure 2A). It was found that the multiple of CP increase in the TN group is slightly higher than that in the TW group. In general, the increase in CP content both in the TN and TW groups indicated that *T. asperellum* can utilize lignocellulose to release some nutrients and obtain energy for its growth and metabolism. The CP content of the TW was higher than that of the TN, because the NaOH pretreatment likely dissolved a large amount of organic matter in the straw cells, some of which are nitrogen-containing organic substances, thus reducing the nitrogen content in the straw (Fang et al., 2019).

The changes in the concentration of reducing sugars during the SSF by *T. asperellum* were shown in Figure 2B. In the TN and TW groups, the concentration of reducing sugars initially increased within the first 5 days and then gradually decreased to stabilize by day 30. On day 5, the maximum concentrations of reducing sugars were 12.41 and 10.86 mg/g, respectively, and by day 30, these values dropped to 9.30 and 6.38 mg/g. Nevertheless, the content of reducing sugars in the TN group is higher than that in the TW group. To further investigate the composition and content of monosaccharides after the fermentation, targeted and semi-quantitative analyses were conducted using HPLC. Monosaccharides related to cellulose and hemicellulose, such as glucose, xylose, galactose, and arabinose, as well as those related to pectin, including mannose, rhamnose, and fucose, were identified (Supplementary Tables S2, S3), indicating that *T. asperellum* possesses a rich array of degradation enzymes. A semi-quantitative analysis of glucose, xylose, galactose, and arabinose was performed, revealing a relatively low glucose content on day 5 (Supplementary Figure S1). This suggested that during the 0–5 day period, *T. asperellum* rapidly utilized glucose from the nutrient solution for growth and continuously degraded lignocellulose, accompanied by the production of a large amount of reductive non-monosaccharides. This accounted for the phenomenon of low glucose concentration and high reducing sugar concentration. As fermentation progressed, the glucose concentration in the TN and TW groups gradually increased, reaching maximum values of 2.28 and 2.51 mg/g on day 30, respectively. This indicates that in the later stages of fermentation, the main process was potentially the slow hydrolysis of oligosaccharide chains into monosaccharides. In addition, the gradual stabilization of reducing sugar concentration suggested that *T. asperellum* is in the balance between degrading lignocellulose and maintaining its own metabolism. The study showed that *T. asperellum* used nitrogen sources to produce degrading enzymes during the lignocellulose degradation process, improving degradation efficiency and raising the substrate’s CP content. The fermented substrate included a considerable number of monosaccharide molecules, suggesting that *T. asperellum* has a large number of polysaccharide-degrading enzymes that efficiently broke down lignocellulose. This enhanced the fermented substrate’s nutritional makeup and validated *T. asperellum*’s strong lignocellulose breakdown ability.

### 3.3 Enzyme activity assay

**Figure 3:**
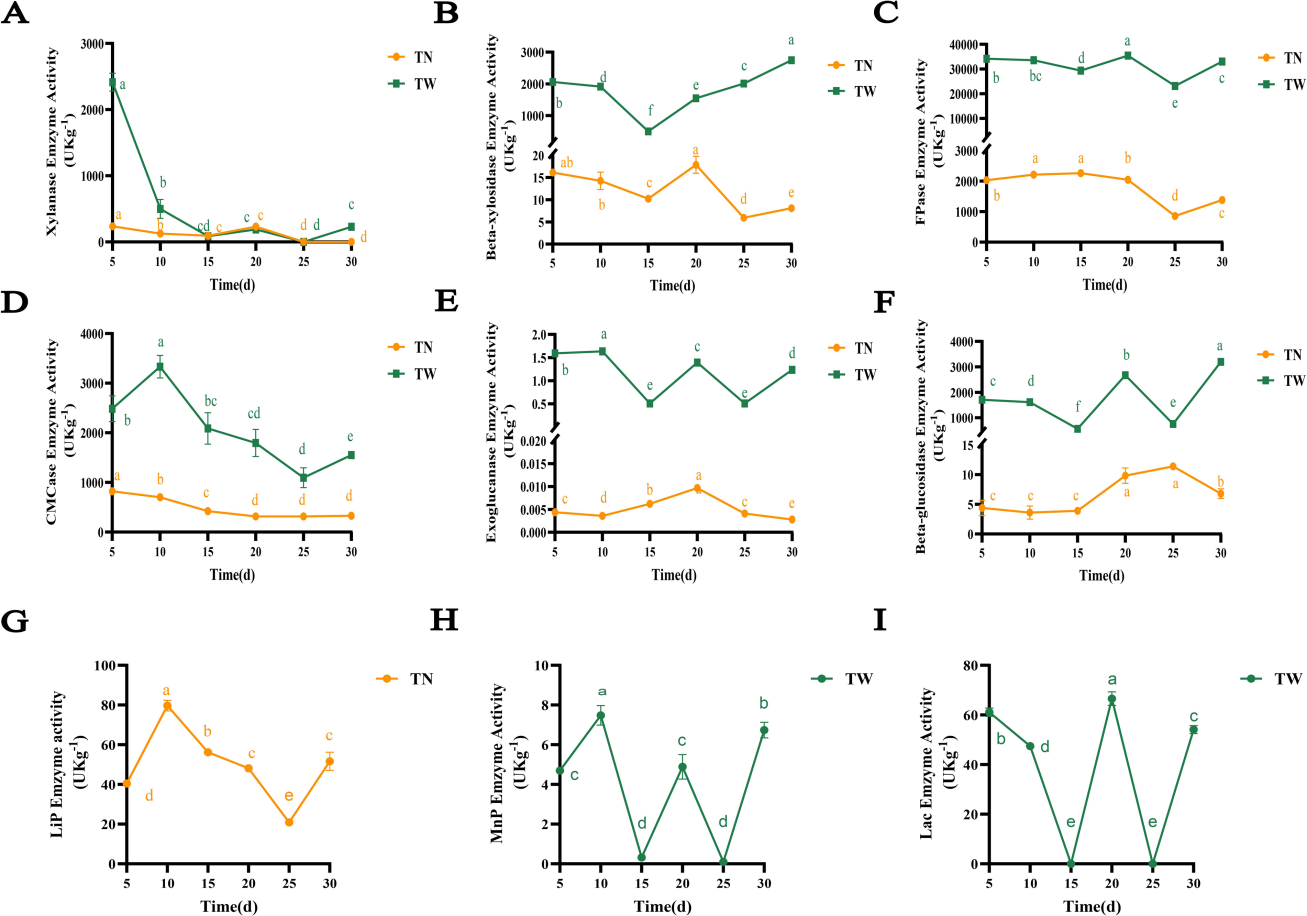
The enzymatic activity of lignocellulose-degrading enzymes produced by *T. asperellum* in the SSF. Different letters indicate significant differences at a *p*-value of < 0.05. **(A)** Xylanase, **(B)** Beta-xylosidase, **(C)** FPase, **(D)** CMCase, **(E)** Exoglucanase, **(F)** Beta-glucosidase, **(G)** LiP, **(H)** MnP, **(I)** Lac.

In SSF, *T. asperellum* produced lignocellulolytic enzymes to degrade WS, and their enzymatic activities changed dynamically over the fermentation period as shown in Figure 3. Xylan is one of the main components of hemicellulose in WS, and the primary decomposing enzymes include xylanase and β-xylosidase, among others. Xylanase refers to a complex of enzymes that degrade xylan. The TW group exhibited the highest xylanase activity on the 5th day, reaching 2412.75 U/kg (Figure 3A). Subsequently, the enzyme activity was gradually decreased and stabilized. Similarly, the TN group had the maximum enzyme activity on the 5th day, reaching 235.90 U/kg, and the activity of this group remained relatively stable after the 5th day until it disappeared on the 25th day. This corresponds to the slow increase in hemicellulose degradation rates from day 5 to 30. The β-xylosidase enzyme activity first decreased and then gradually increased, reaching the maximum on the 30th day at 2744.91 U/kg in the TW group (Figure 3B). As fermentation progressed, it is speculated that the accumulation of xylooligosaccharides increased, potentially necessitating the production of more β-xylosidase. The β-xylosidase activity of the TN group was observed to decrease initially, subsequently increase, and then gradually decline again. The TN group had the maximum enzyme activity on the 20th day at 17.87 U/kg.

Cellulose degradation often relies on FPase, CMCase, exoglucanases, and β-glucosidases as shown in Figures 3C–F. The FPase reflected the total cellulase enzymatic activity in SSF. The TW group exhibited a dynamic change in FPase activity, decreasing and then increasing before decreasing again, with the maximum activity of 35413.25 U/kg observed on the 20th day (Figure 3C). In contrast, the TN group showed a relatively steady rise in FPase activity during the first 15 days, reaching its peak at 2263.03 U/kg. CMCase primarily hydrolyzes β-1,4-glycosidic bonds in amorphous cellulose to release cellulose oligosaccharides. Similarly, in SSF, its enzyme activity first increased and then decreased before rising again, with the highest activity of 3333.22 U/kg on the 10th day (Figure 3D). In contrast, the TN group showed a relatively flat change in CMCase activity, with the maximum activity of 822.38 U/kg on the 5th day. Exoglucanases mainly act on the ends of cellulose polysaccharide chains, hydrolyzing β-1,4-glycosidic bonds and releasing cellobiose molecules, hence also known as cellobiase. Like CMCase and FPase, the exoglucanase activity in the TW group varied significantly over the fermentation period (Figure 3E). The highest enzyme activity was observed on the 5th day at 1.59 U/kg. Similarly, the exoglucanase activity in the TN group was initially increasing and subsequently declining, peaking at 0.01 U/kg on the 20th day. Beta-glucosidase is responsible for hydrolyzing cellobiose and oligosaccharide carbohydrates into glucose. The β-glucosidase activity in the TW group also changed significantly over the fermentation period (Figure 3F). The enzyme activity increased and reached maximum concentration of 3196.77 U/kg at the final stage of fermentation, suggesting that as fermentation progressed, the production of oligosaccharide chains might have increased, potentially leading to an increase in the enzyme activity. Similarly, the enzyme activity in the TN group demonstrated a pattern of gradual increase followed by a subsequent decline. Which slowly rose from the 15th day until the highest value of 11.40 U/kg was observed on the 25th day. The dynamic changes in enzymes activity also reflected the utilization of lignocellulose in WS by *T. asperellum*, which is consistent with its increase of cellulose degradation rate.

The types of lignin-degrading enzymes secreted by *T. asperellum* varied with the species of substrate as shown in Figures 3G–I. In the TN group, only LiP was detected, which initially increased and then decreased before rising again, reaching a maximum concentration of 79.10 U/kg on the 10th day (Figure 3G). However, LiP activity was not detected, Lac and MnP activities were present, with significant changes during the fermentation in the TW group. The maximum activities of Lac and MnP were observed on the 20th day (66.59 U/kg) and the 10th day (7.48 U/kg), respectively (Figures 3H,I). The dynamic enzyme activities not only directly affect the efficiency of lignocellulose degradation but also provide a basis for transcriptomic studies through the elucidation of gene expression patterns.

### 3.4 SEM analysis

**Figure 4:**
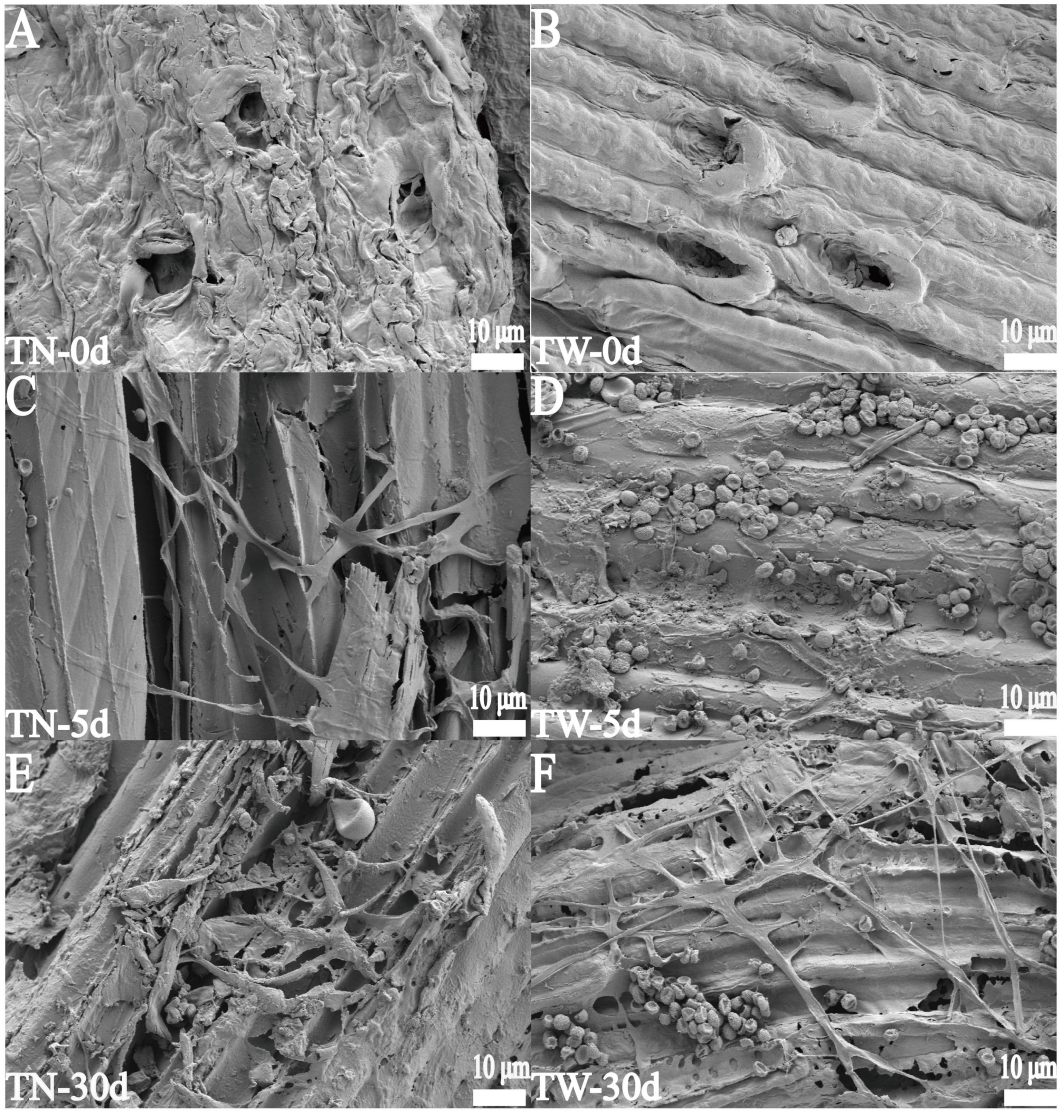
The SEM images of WS during SSF. **(A, C, E)** represent 0, 5, and 30 days in the TN group, respectively; **(B, D, F)** represent 0, 5, and 30 days in the TW group, respectively.

In the TN group, the WS pretreated with NaOH exhibited surface structural changes due to the cleavage of covalent bonds between lignin and hemicellulose. The surface became less smooth, with an increased number and size of pores and wrinkles, which facilitated hyphal extension and penetration (Figure 4A). In contrast, the surface structure of the original WS in the TW group remained relatively smooth and compact (Figure 4B). On day 5 of fermentation, hyphae and spores were observed covering the surface of the WS. In the TN group, the hyphae were more capable of penetrating and disrupting the surface of the WS tissue during the same fermentation period (Figure 4C). In the TW group, only a few hyphae penetrated the surface (Figure 4D). By day 30, the originally compact wheat straw tissue structure in both the TN and TW groups had become looser, with larger pores and a more relaxed structure. Hyphae traversing the large gaps within the straw tissue were clearly visible, causing varying degrees of mechanical damage to the straw tissue and promoting its degradation, as shown in Figures 4E,F, respectively. Comparative analysis of WS surfaces structures before and after fermentation via SEM revealed that *T. asperellum* penetrated the straw matrix through hyphal networks, accompanied by the secretion of lignocellulose-degrading enzymes. This resulted in distinct surface fissures and the formation of porous structures within the substrate. The synergistic effect between physical structural disruption and enzymatic hydrolysis conclusively demonstrates the degradative capacity of *T. asperellum* toward lignocellulosic composite substrates.

### 3.5 FTIR analysis

**Figure 5:**
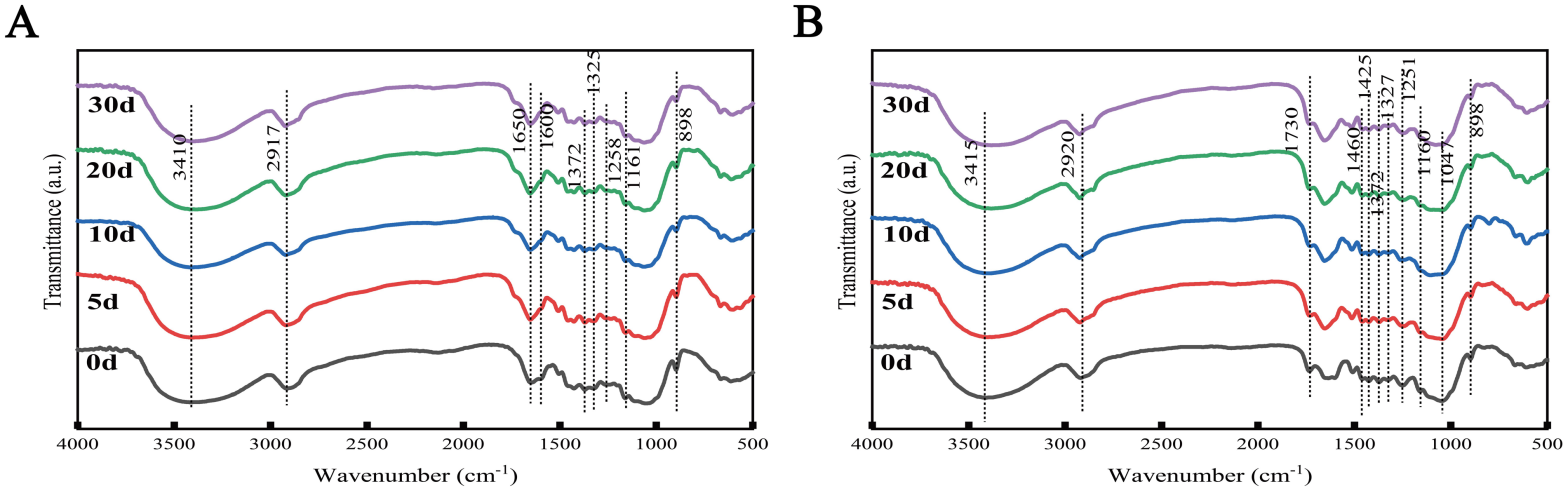
The FTIR spectra of TN **(A)** and TW **(B)**.

The FTIR characteristic absorption peaks of the TN and TW groups during SSF were shown in Figure 5. FTIR analysis was used to determine the changes in lignocellulose over the fermentation period. It summarized the chemical bond functional groups corresponding to the peak positions in the FTIR spectra of the TN and TW groups (Supplementary Tables S4, S5), as well as the relative percentage changes in peak intensity compared to day 0. After fermentation by *T. asperellum*, even though the peak positions of different lignocelluloses at various degradation stages were roughly the same, their peak intensities varied. In the TN group, the chemical bonds related to lignocellulose showed increasingly strong changes with the advancement of SSF. A band at around 3,400 cm^−1^ (O-H stretching), related to cellulose, was observed; a band at around 2,917 cm^−1^ (C-H stretching) was attributed to cellulose and hemicellulose (Xu et al., 2013). Bands at 1650 and 1,600 cm^−1^ (C=C stretching) were assigned to the aromatic skeleton of lignin (Faix, 1991; Bahng et al., 2011). Bands at 1372 cm^−1^ (C-H stretching), around 1,325 cm^−1^ (C-O guaiacyl ring stretching), and around 1,258 cm^−1^ (C-O ether stretching) were also assigned to lignin (Xu et al., 2013). The band at around 1,061 cm^−1^ (C-O-C asymmetric stretching) between 1,200 and 1,035 cm^−1^ is related to the stretching of cellulose and hemicellulose (Kumar et al., 2009). The band at 898 cm^−1^ (C-H), characteristic of the β-(1,4) glycosidic linkages in amorphous cellulose, was also identified (Zhao et al., 2013). The relative changes of these chemical bonds were highest on the 30th day, indicating that with the advancement of fermentation, *T. asperellum* degraded lignocellulose more thoroughly, demonstrated the ability of *T. asperellum* to degrade lignocellulose in SSF. For the TW group, in addition to the peak positions corresponding to the same chemical bonds as the TN group, the band at 1730 cm^−1^ (C=O) attributed to acetyl groups, which are associated with their conjugates (e.g., xylans), was best detected in the hemicellulose, and this band weakened and failed to form a peak in the NaOH-pretreated WS, consistent with the destruction and reduction of hemicellulose content in the straw after NaOH pretreatment. The band at 1047 cm^−1^ (C-O stretching) is related to cellulose and hemicellulose. In the TW group, only the relative changes in cellulose-related bonds at 1160 cm^−1^, 1,047 cm^−1^, and 898 cm^−1^ were positive, while the rest were negative, indicating no change compared to day 0, which is consistent with the increase of cellulose degradation rate of the TW group. The same phenomenon was also observed after NaOH pretreatment. Compared to day 0, the bands at 1160 cm^−1^, 1,047 cm^−1^, and 898 cm^−1^ all showed different degrees of increased intensity after fermentation, while the bands related to lignin showed no change, consistent with the increase of cellulose degradation rate and the decrease of lignin degradation rate of the TW group. FTIR analysis revealed a significant reduction in the intensity of characteristic absorption peaks associated with lignocellulose (e.g., C=C stretching vibration peaks of lignin aromatic rings and β-glycosidic bond vibrations in cellulose) after fermentation. This reduction can be attributed to the selective cleavage of specific chemical bonds in the substrate by lignocellulose-degrading enzymes secreted by *T. asperellum*, thereby resulting in the weakening of corresponding absorption peaks.

### 3.6 Transcriptome analysis

#### 3.6.1 Transcriptional profiling of *Trichoderma asperellum* during SSF

**Figure 6:**
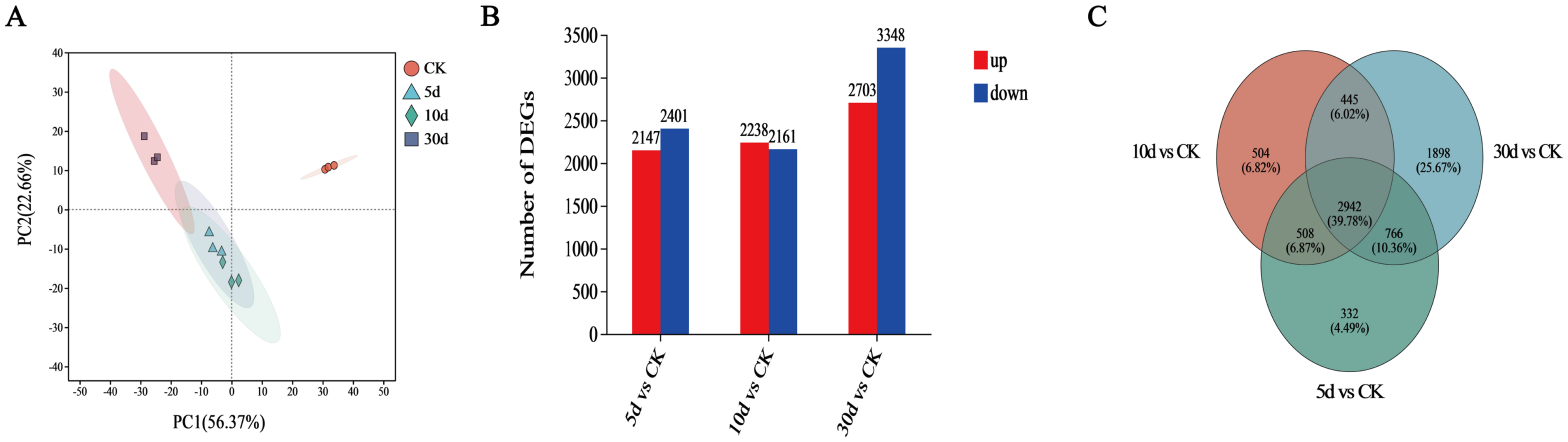
Transcriptome analysis of *T. asperellum*. **(A)** Principal component analysis (PCA). **(B)** Number of DEGs. **(C)** Venn diagram.

The fundamental reasons for selecting *T. asperellum* from the TN group for transcriptome analysis lies in the fact that after NaOH pretreatment, a portion of the macromolecular lignocellulosic fragments are degraded into smaller fragments, thereby increasing the diversity of substrates (Lobo et al., 2021). We believe the increase in substrate diversity may be favorable for inducing the expression of many key genes in *T. asperellum*. In addition, to characterize the gene expression profiles of *T. asperellum* at different cultivation times, a transcriptome analysis was conducted to identify DEGs. PCA analysis indicated that the 12 samples could be distinctly assigned to four groups as shown in Figure 6A. It was observed that the three samples from each group were all within their respective confidence circles, indicating good repeatability among samples within the same group. A total of 12,801 expressed genes were detected in this analysis. Compared with the CK, 4548 DEGs (up 2,147, down 2,401), 4,399 DEGs (up 2,238, down 2,161) and 6,051 DEGs (up 2,703, down 3,348) were identified on day 5, 10, and 30, respectively as shown in Figure 6B. Compared to the CK, there are a total of 2,942 DEGs at the intersection (Figure 6C). The above mentioned DEGs were further analyzed.

#### 3.6.2 GO analysis and key genes of lignocellulose

**Figure 7:**
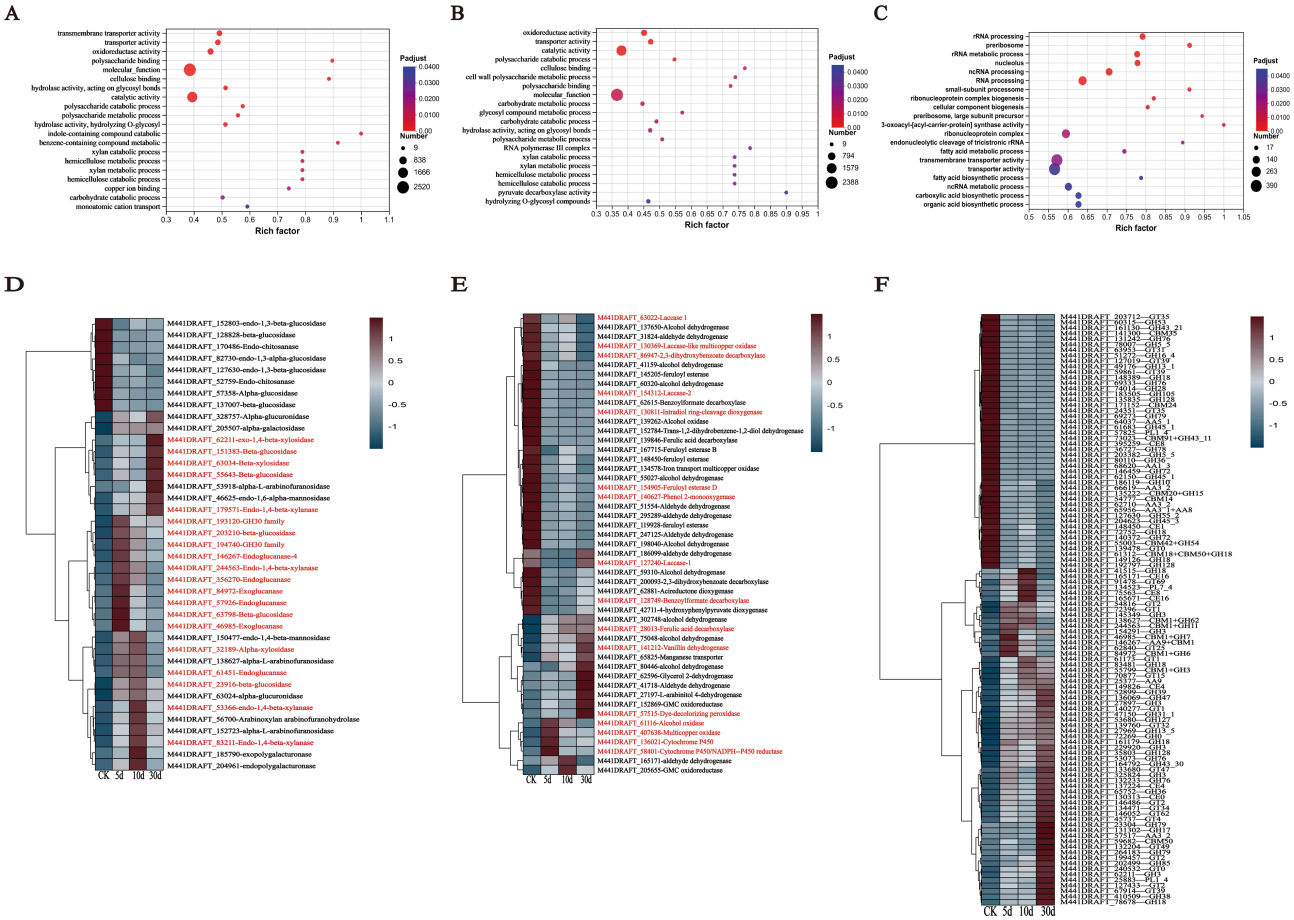
GO enrichment terms and cluster analysis heatmap of *T. asperellum*. **(A–C)** Represent the 20 GO terms enriched by the DEGs of *T. asperellum* on days 5, 10, and 30, respectively. The GO enrichment items are based on the adjusted *p*-value criteria. **(D,E)** Show the expression levels of DEGs involved in carbohydrate metabolism and lignin metabolism. **(F)** Show a heatmap of DEGs annotated by the CAZy database. The DEGs presented in the heatmap are differentially expressed in pairwise comparisons between days 5, 10, and 30, and the fungus grown on PDA for 5 days. Gene names in red letters indicate specific contents discussed in the text.

To further explore the potential pathways expressed by this fungus during SSF, we conducted GO enrichment analysis on the upregulated and downregulated genes from each transcriptome comparison. The GO analysis revealed that *T. asperellum* expressed distinct biological functions at different days of growth compared to the CK as shown in Figures 7A–C. In pairwise comparisons with the CK, the growth of *T. asperellum* on the 5th and 10th days of SSF typically enriched GO terms related to cellulose and hemicellulose degradation including carbohydrate catabolism, carbohydrate transport, glycoside hydrolases, polysaccharide metabolism, and hemicellulose and xylan catabolic processes, as well as oxidoreductases, copper ion binding, and phenolic compound metabolism processes associated with lignin degradation. On the 30th day, the primarily enriched GO terms were processes related to RNA and rRNA transcription, which are more associated with the growth and development of *T. asperellum*.

The genes associated with cellulose and hemicellulose carbohydrate metabolism were shown in Figure 7D. In the cellulose metabolism pathway, DEGs encoding endoglucanase (gene-M441DRAFT_146267, gene-M441DRAFT_356270, gene-M441DRAFT_57926, gene-M441DRAFT_61451) were all upregulated, particularly on days 5 and 10, indicating that endoglucanase is positioned at the initial stage of cellulose degradation, which correlates with the higher enzyme activity observed during the early stages of fermentation. DEGs encoding exoglucanase (gene-M441DRAFT_46985, gene-M441DRAFT_84972) were also upregulated. Genes encoding β-glucosidase (gene-M441DRAFT_151383, gene-M441DRAFT_203210, gene-M441DRAFT_23916, gene-M441DRAFT_55643, gene-M441DRAFT_63798) were upregulated as well. It is consistent with the increase of related cellulases activity and the decrease of cellulose content in SSF. In the hemicellulose metabolism pathway, genes encoding xylanase (gene-M441DRAFT_179571, gene-M441DRAFT_193120, gene-M441DRAFT_194740, gene-M441DRAFT_244563, gene-M441DRAFT_53366, gene-M441DRAFT_83211) were upregulated during SSF, facilitating the conversion of xylan to xylooligosaccharides and xylobiose. Genes encoding β-xylosidase (gene-M441DRAFT_32189, gene-M441DRAFT_62211, gene-M441DRAFT_63034) were also upregulated during SSF, promoting the release of xylose monomers from oligosaccharides. It is consistent with the increase of related hemicellulases activity and the decrease of hemicellulose content in SSF. Not only that, the genes expressing α-L-arabinofuranosidase and α-galactosidase were up-regulated in SSF, indicating that *T. asperellum* can secrete these enzymes to degrade hemicellulose. With the upregulation of α-mannanase and α-galactosidase gene expression, it indicated that *T. asperellum* is capable of secreting these enzymes to degrade pectin components, which is also consistent with the monosaccharides results detected by HPLC. Additionally, genes associated with fungal growth and development were downregulated, such as endo-chitosanase and endo-1,3-β-glucanase, enzymes related to the synthesis of fungal cell wall polysaccharides.

The degradation of lignin primarily involves converting the polymeric macromolecule lignin into smaller molecular fragments. This process necessitates lignin-modifying enzymes, such as Lac, MnP, Lip, and dye-decolorizing peroxidase (DyP) (Becker and Wittmann, 2019). Specifically, gene-M441DRAFT_127240 (Lac-1), gene-M441DRAFT_63022 (Lac 1), and gene-M441DRAFT_154312 (Lac-2) were downregulated in SSF, which correlates with the absence of Lac activity detected in the TN group of *T. asperellum*. Interestingly, compared to the CK, gene-M441DRAFT_57515 (DyP) exhibited consistently upregulated expression during SSF, suggesting that *T. asperellum* can secrete DyP to modify lignin and reduce its content (Figure 7E). Additionally, lignin-degrading auxiliary enzymes are required, which primarily participate indirectly in lignin degradation by producing hydrogen peroxide through the oxidation of small molecular substrates (Bugg et al., 2020). For instance, gene-M441DRAFT_61116 (Alcohol oxidase) and gene-M441DRAFT_407638 (Multicopper oxidase) were both upregulated during SSF. The expression of these two oxidases was higher on days 5 and 10. Then, these small molecular fragments of aromatic compounds were further degradation, including demethylation, hydroxylation, decarboxylation, and dioxygenase-mediated ring-opening (Erickson et al., 2022). The cytochrome P450 family can participate in demethylation reactions (Perez et al., 2021). The related genes including gene-M441DRAFT_136021 (Cytochrome P450 55A2) and gene-M441DRAFT_58401 (Cytochrome P450/NADPH--P450 reductase) were both upregulated during SSF, with the highest expression observed on day 5, indicating that cytochrome P450 was involved in the demethylation of aromatic compounds during the early fermentation stage. Gene-M441DRAFT_140627 (Phenol 2-monooxygenase fsqG) is considered a catalyst for the hydroxylation of aromatic compounds (Sucharitakul et al., 2005). Its expression was downregulated compared to the CK. In the decarboxylation reaction, gene-M441DRAFT_86947 (2,3-dihydroxybenzoate decarboxylase) was downregulated compared to the CK (Kamath et al., 1989). In the case of dioxygenase gene-M441DRAFT_130811 (Intradiol ring-cleavage dioxygenase hqdA) exhibited downregulated expression compared to the CK. The downregulation of the enzymes genes involved in the hydroxylation, decarboxylation, and ring-opening reactions may be key to limiting the degradation of lignin by *T. asperellum*. Additionally, related lignin aromatic compounds degradation genes, such as gene-M441DRAFT_28013 (Ferulic acid decarboxylase 1) and gene-M441DRAFT_141212 (Vanillin dehydrogenase), were found to be upregulated, indicating that these enzymes were primarily involved in the conversion of ferulic acid to vanillin and ultimately to vanillic acid.

**Figure 8:**
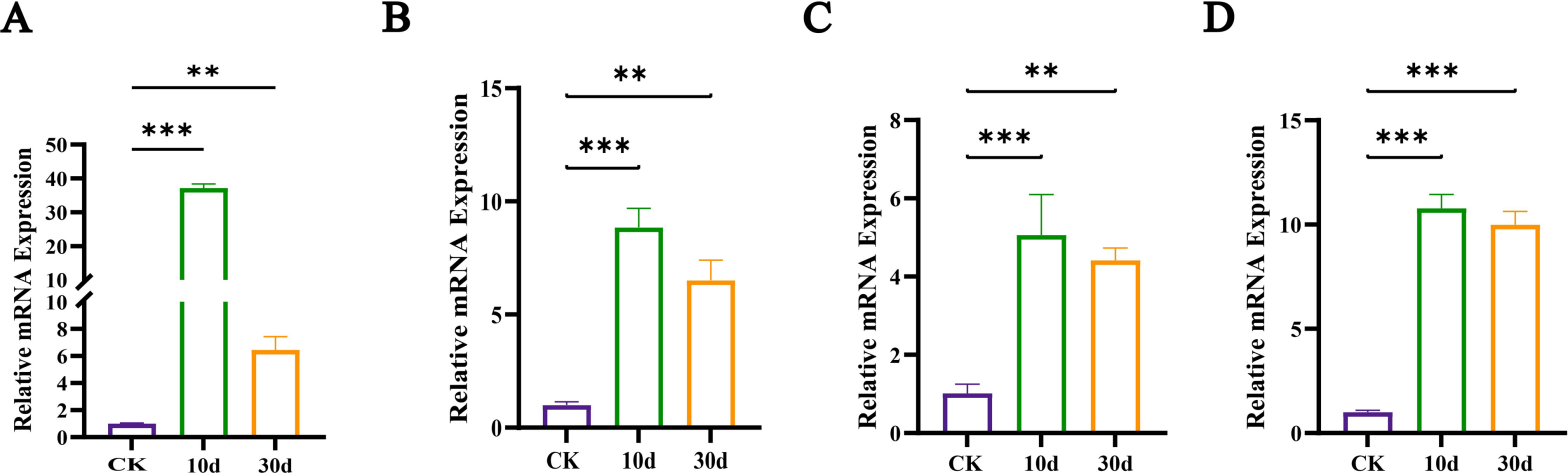
Relative mRNA expression abundance of genes involved in lignocellulosic degradation. **(A)** The gene M441DRAFT_83211, **(B)** the gene M441DRAFT_61279, **(C)** the M441DRAFT_193120, **(D)** the gene M441DRAFT_14626. ** and *** indicate *p* < 0.05 and *p* < 0.01, respectively.

We performed a comparison of the 2,942 shared DEGs against the CAZy database and identified 536 DEGs with annotations as shown in Figure 7F. These included 292 glycoside hydrolases (GHs, enzymes that hydrolyze glycosidic bonds in polysaccharides), 101 glycosyltransferases (GTs, which transfer sugar moieties from donors to acceptors and disrupt glycosidic bonds in various substrates), 57 carbohydrate-binding modules (CBMs, which bind to carbohydrates), 51 auxiliary activity enzymes (AAs, involved in lignin degradation and mineralization), 24 carbohydrate esterases (CEs, which hydrolyze carbohydrate substrates by removing ester groups), and 11 polysaccharide lyases (PLs, which cleave non-hydrolyzable glycosidic bonds). The heatmap above displayed the top 20% of DEGs by expression variance (Figure 7F), including genes related to cellulose degradation (GH1, GH2, CBM1, and AA9), hemicellulose degradation (GH5, GH30, and GH31), and lignin degradation (AA1). Thus, the expression of certain cellulose and hemicellulose-degrading enzyme systems in *T. asperellum* was induced and upregulated, such as endoglucanases, exoglucanases, β-glucosidases, xylanases, and β-xylosidases, which is consistent with the increase of corresponding enzymes activities and the reduction in lignocellulose content. Meanwhile, *T. asperellum* expressed more genes related to lignocellulosic degrading enzymes, which also reflected the strong lignocellulosic degrading ability. While the ability of *T. asperellum* to degrade lignin may have been underestimated, it does not imply that *T. asperellum* lacks the capacity to degrade lignin. To validate the transcriptome data, RT-qPCR was performed for four lignocellulose-related and large expression difference DEGs, and these four genes were derived from the shared DEGs. Their expression trends were largely consistent with those observed in the transcriptomic analysis, all showing upregulation compared to the CK. This confirmed the concordance of the RT-qPCR results with the transcriptomic analysis outcomes (Figure 8).

## 4 Discussion

### 4.1 The ability of *Trichoderma asperellum* to degrade lignocellulose

In this study, the content of CF is negatively correlated with the palatability of straw as feed, meaning that the lower the CF content, the better the palatability (Wang et al., 2022). Therefore, after fermentation by *T. asperellum*, the reduction in CF content increases the potential for the fermented WS to be used as feed. *T. asperellum* significantly altered the lignocellulose content in both TW and TN groups, particularly reducing the content of hemicellulose and cellulose, indicating that *T. asperellum* has pathways to degrade them to maintain its own growth and metabolism. This is compared to Shrivastava’s experiment (Shrivastava et al., 2012), where SSF of WS with *Ganoderma* sp. resulted in degradation rates of 22.6 and 21.73% for cellulose and hemicellulose, respectively, after 10 days of fermentation. In comparison, *T. asperellum* demonstrated superior performance in the utilization of cellulose and hemicellulose from WS. Furthermore, *Schizophyllum commune* exhibited degradation efficiencies of 30–40% in finger millet straw, 27–32% in rice straw, 21% in WS, and 26% in corn straw (Kumar et al., 2022). These data collectively highlight the superior performance of *T. asperellum* in utilizing cellulose and hemicellulose within WS, underscoring its enhanced lignocellulose-degrading capability compared to other fungal species under similar conditions. Interestingly, it may be due to the increase in the content of crystalline cellulose in WS after NaOH pretreatment, which made it more difficult for *T. asperellum* to disperse and utilize (Berhe et al., 2023), hence the higher cellulose degradation rate in the TW group compared to the TN group. Similarly, we observed an increasing trend in lignin content in both the TW and TN groups, and a corresponding decreasing trend in the lignin degradation rate. This is probably due to the strong capabilities of *T. asperellum* in degrading cellulose and hemicellulose, which can result in increasing the relative content of lignin (Popescu et al., 2007). Secondly, the Van Soest method represents the total lignin content with acid-insoluble lignin, thus changes in acid-soluble lignin content cannot be reflected.

### 4.2 The lignocellulose-degrading enzymes capability of *Trichoderma asperellum*

Through enzyme activity assays, it was found that *T. asperellum* has a rich and potent array of hemicellulose and cellulose degrading enzymes, even stronger than other soft-rot fungi known for their cellulose and hemicellulose degradation capabilities. *Pestalotiopsis microspora* exhibited lower FPase activity of 13 U/g after 6 days of fermentation on wheat bran compared to the activity in the TW group of *T. asperellum* on day 5 (Goukanapalle et al., 2020). Another study reported that using *Trichoderma guizhouense* and *T. reesei* for SSF on WS, respectively, the CMCase and β-glucosidase were both less than 1 U/mL on the fifth day (Grujić et al., 2019). Wang et al. (2017) reported that the maximum FPase activities of *T. asperellum* T-1 and *T. reesei* QM6a during liquid-state fermentation of corn straw for 16 days were both below 1 U/mL and were not significantly different from each other. Lever et al. (2010) reported that when fermenting WS with *T. reesei*, the FPase activity was below 1 U/g on both day 10 and day 14. Consequently, *T. asperellum* not only has a more comprehensive cellulase system but also exhibits stronger cellulolytic enzyme activity. Additionally, the presence of lignin-degrading enzyme systems in *T. asperellum*, including Lac, MnP, and LiP, indicates that *T. asperellum* indeed has the capability and potential to degrade lignin, making it a potential candidate strain for the production of lignocellulosic degrading enzymes preparations. The reason for the absence of Lac and Mnp in the TN group is that the three-dimensional structure of lignin is altered after NaOH pretreatment, particularly the destruction of the phenolic end aromatic ring structures, thus preventing the production of enzymes like Mnp and Lac, which have a strong oxidative capacity toward phenolic lignin (Higuchi, 2004). However, LiP activity was not detected in the TW group, possibly due to competition between Mn^2+^ and Mn^3+^ for the active site of LiP, inhibiting its activity (Zhang S. et al., 2020; Zhang S. et al., 2022). Therefore, even the same type of substrate with different structures induces different types and activities of lignin-degrading enzymes (Shi et al., 2021). The determination of enzyme activities revealed that *T. asperellum* possesses the expression genes related to lignin-degrading enzymes such as Lac, MnP, and LiP, in other words, it is a soft-rot fungus with the potential to degrade lignin, filling the gap in the understanding of *T. asperellum*’s role in lignin degradation. Furthermore, it was found that the enzymes activities of the TW group were generally higher than those of the TN group. This is consistent with Wang Quan’s conclusion that, after SSF of rice straw treated with *T. asperellum* under acidic pretreatment, alkaline peroxide pretreatment, and untreated conditions, respectively (Wang et al., 2017). Based on the enzyme activity alone, it indicates that *T. asperellum* is more suitable for degrading natural lignocellulose, as it may possess a more comprehensive set of endogenous lignocellulolytic enzymes, thus showing excellent enzyme activity expression even in untreated WS fermentation. Finally, it was found that the variation of the enzymes activity in TN group was generally more stable than that in TW group. This is because the NaOH pretreatment altering the structure and content of lignocellulose, leading to the appearance or increase of some oligosaccharides, thus enriching the types of fermentation substrates, and therefore the enzyme activity remained relatively stable, unlike the untreated WS group, where the appearance of one or a few substrates would induce a rapid increase in degrading enzymes (Siripong et al., 2018).

### 4.3 Changes of nutrient compositions and structure of WS before and after fermentation

It was observed that the CP content of the TN group on the 5th day was significantly higher than that on the 10th day, with no significant difference from the CP content on the 20th day. It is possible that the CP after fermentation includes not only fungal proteins, but also extracellular proteins produced (such as lignocellulolytic enzymes), and some nitrogen-containing metabolites. Among these nitrogen-containing metabolites (ammonia nitrogen metabolites), some will volatilize and be lost (Tao et al., 2020), hence the CP content decreases but with no significant difference. Similarly, the TW group also exhibited a similar phenomenon, mainly from 10 to 30 days. SEM and FTIR spectroscopy were used to analyze the physical and chemical structural characteristics of WS before and after fermentation. *T. asperellum*, by secreting a lignocellulose-degrading enzyme system and through the mechanical forces of hyphal attachment and penetration, caused the damage of the surface structure of the straw and the disruption of characteristic covalent bonds. This is consistent with previous reports (Li et al., 2020; Zhou et al., 2024). Previous studies by Wang et al. (2023) demonstrated that solid-state fermentation of corn straw and rice straw with *Phanerochaete chrysosporium*, optimized by supplementing exogenous carbon sources, yielded maximum CP contents of only 3.90 g/kg (corn straw) and 3.68 g/kg (rice straw) after 21 days of fermentation. In contrast, *T. asperellum* in this study exhibited significantly higher CP enrichment in WS, highlighting its unique advantages in lignocellulose degradation and nutrient conversion.

Notably, the net accumulation of reducing sugars in the TN group exceeded that in the TW group. This discrepancy may stem from strain-specific metabolic preferences: the TW group exhibited higher lignocellulose-degrading enzyme activity, necessitating greater consumption of substrate sugars (e.g., glucose) for enzyme biosynthesis and energy metabolism, thereby reducing net residual sugar. Conversely, the TN group achieved a balanced coordination between enzymatic activity and substrate utilization, enabling efficient lignocellulose degradation while retaining higher levels of reducing sugars. In contrast, *Aspergillus ochraceus* and *A. terreus* yielded merely 11.9 mg/g and 10.1 mg/g of reducing sugars, respectively, after 5 days of rice straw decomposition at 25°C, which are substantially lower than the reducing sugar production observed in the TN group with *T. asperellum* (Helal, 2005). These comparative data further corroborate the superior capability of *T. asperellum* in utilizing complex lignocellulosic substrates.

### 4.4 The degradation pathways of lignocellulose by *Trichoderma asperellum*

**Figure 9:**
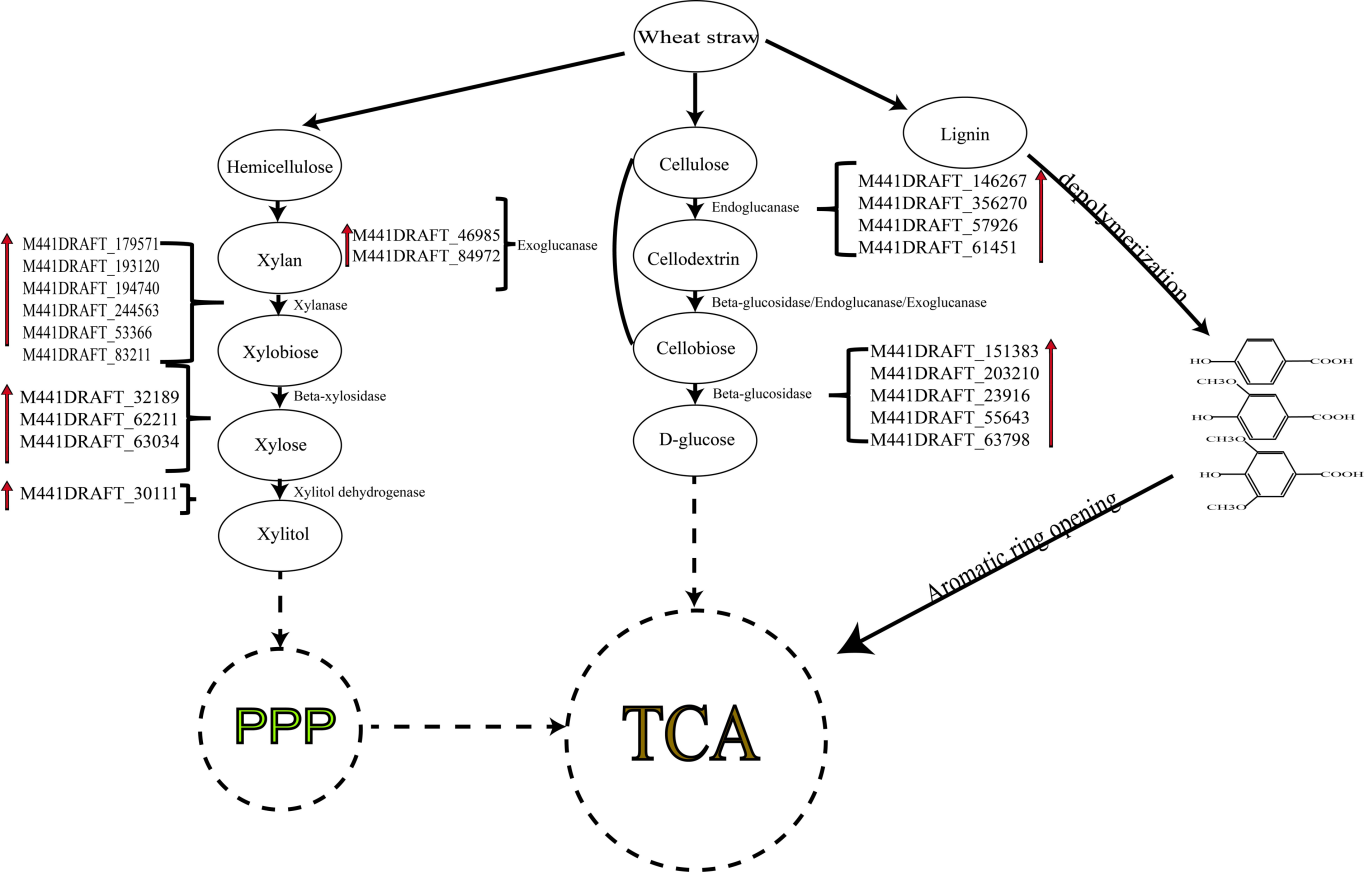
Schematic representation of the lignocellulose degradation pathways by *T. asperellum*.

Through transcriptome analysis, it was discovered that *T. asperellum* can express DyP, which is responsible for the modification of lignin, this is a novel finding. Hemicellulose and cellulose are degraded stepwise by relevant enzymes from polysaccharides to oligosaccharides and then to monosaccharides, which eventually enter the tricarboxylic acid (TCA) cycle through biochemical reactions for material metabolism and energy conversion (Figure 9). Under the catalysis of related degrading enzymes, cellulose is converted into cellodextrins, then into cellobiose, and finally into glucose, which enters the TCA cycle. Similarly, xylan in hemicellulose is broken down into xylobiose and then into xylose monomers. Xylose is transformed into xylitol by xylose dehydrogenase (gene-M441DRAFT_30111) and enters the pentose phosphate pathway (PPP) before entering the TCA cycle to provide energy for microbes. For lignin metabolism, the main process involves the depolymerization of lignin into lignin monomers such as 4-hydroxybenzoic acid, protocatechuic acid, and gallic acid. These compounds undergo ring-opening reactions and enter the β-ketoglutarate pathway before finally entering the TCA cycle.

Although this study offered initial insights into *T. asperellum*’s efficient degradation of WS lignocellulose and identified DEGs related to lignocellulosic enzyme systems through transcriptomic analysis, there are limitations to address in future work. First, the degradative functions of these genes have not been confirmed experimentally, and their role as central regulatory genes remains unclear. Also, the lack of multi-omics data means the connection between gene expression, protein function, and metabolite pathways has not been established. Furthermore, although lignocellulose-degrading enzyme activities were detected in both the TW and TN groups of *T. asperellum*, the study did not compare specific or relative enzyme activities with model strains under the same conditions. Therefore, it is difficult to assess its industrial application potential.

In summary, this study dynamically explored the degradation of lignocellulose in various wheat straws by *T. asperellum* over different fermentation periods. Results revealed that *T. asperellum* is a fungus with a rich lignocellulolytic enzyme system and abundant carbohydrate-active enzyme gene expression. Based on these findings, this research offers valuable insights for potential applications and future directions in the field. The efficient lignocellulose-degrading ability of *T. asperellum* makes it a strong candidate for biofuel and bio-based chemical production, supporting sustainable energy solutions such as fertilizers, feed, or bioethanol. Additionally, the detailed understanding of its enzyme system and gene expression provides a foundation for further genetic engineering efforts aimed at enhancing its degradation capabilities. Future research could focus on exploring the synergistic effects of combining *T. asperellum* with other microbial species to improve the overall efficiency of biomass conversion processes. Furthermore, optimizing fermentation conditions and scaling up the fermentation process could help realize the industrial application potential of *T. asperellum*, potentially leading to more effective and environmentally friendly methods for lignocellulosic biomass utilization.

## 5 Conclusion

This study utilized *T. asperellum* for SSF of WS and WS pretreated with NaOH, revealing its degradation of lignocellulose and the key genes involved in the degradation process. From a cost-saving perspective, *T. asperellum* is more capable of degrading and utilizing natural biomass by secreting potent lignocellulolytic enzyme activities to break it down. In terms of the enrichment of nutritional components after fermentation, compared to the TW group, the TN group showed a higher content of fermented reducing sugars and a greater multiple of CP increase. Another benefit of pretreatment was reducing fermentation product loss by *T. asperellum*. *T. asperellum* can also secrete a full suite of lignocellulolytic enzyme systems, including hydrolases, esterases, lyases, auxiliary oxidoreductases, lignin-modifying peroxidases, and peroxidases, thus making it a potential candidate strain for enzyme preparation.

## Data Availability

The datasets presented in this study can be found in the online repository. The name of the repository and accession number(s) are as follows: PRJNA1173924 (NCBI).
