# Supplementary material for: The potential of Trichoderma asperellum for degrading wheat straw and its key genes in lignocellulose degradation
Source: Front Microbiol. 2025 Apr 23;16:1550495. doi: 10.3389/fmicb.2025.1550495 (PMC12055841; doi:10.3389/fmicb.2025.1550495)
Supplement: Supplementary file 1 [file Data_Sheet_1.docx]

**Supplementary Material**

| Table S1 Pimers sequences for RT-qPCR. | |
| --- | --- |
| Gen name | Primer sequence |
| M441DRAFT_83211 | Forward: Gatttgccgtcaactggtct  Reverse: ggcggaaggatatccaaagt |
| M441DRAFT_61279 | Forward:ccaactcggttgttcaaggt  Reverse: attgccatatccctcagcag |
| M441DRAFT_193120 | Forward: tatctcgcttttgggtgctt  Reverse: tctttggcttggaatccatc |
| M441DRAFT_146267 | Forward: atgtcacaagaatgccacca  Reverse: tatccacagtctcgcatcca |

| Table S2 Composition of monosaccharides of the TN group. | | | | | |
| --- | --- | --- | --- | --- | --- |
| Monosaccharides | Time (d) | | | | |
|  | 0 | 5 | 10 | 20 | 30 |
| Mannose | + | + | + | + | + |
| Ribose | + | + | + | + | + |
| Rhamnose | + | + | + | + | + |
| Glucuronic acid | + | + | + | + | + |
| Galacturonic acid | - | - | - | - | - |
| Glucose | + | + | + | + | + |
| Galactose | + | + | + | + | + |
| Xylose | + | + | + | + | + |
| Arabinose | + | + | + | + | + |
| Fucose | + | + | + | + | + |
| + indicates that the ingredient is present, - indicates that the ingredient is not present. | | | | | |

Table S3 Composition of monosaccharides of the TW group.

| Monosaccharides | Time (d) | | | | |
| --- | --- | --- | --- | --- | --- |
|  | 0 | 5 | 10 | 20 | 30 |
| Mannose | + | + | + | + | + |
| Ribose | + | + | + | + | + |
| Rhamnose | + | + | + | + | + |
| Glucuronic acid | + | + | + | + | + |
| Galacturonic acid | + | - | - | - | + |
| Glucose | + | + | + | + | + |
| Galactose | + | + | + | + | + |
| Xylose | + | + | + | + | + |
| Arabinose | + | + | + | + | + |
| Fucose | + | + | + | + | + |
| + indicates that the ingredient is present, - indicates that the ingredient is not present. | | | | | |

| Table S4 Assignment of the peaks to functional groups, biomass components and their percentage changes relative due to pretreatments by FTIR in TN. | | | | | | |
| --- | --- | --- | --- | --- | --- | --- |
| Wave number (cm^-1^) | Asignment | Biomass component | Intensity (% relative percentage changes) | | | |
|  |  |  | 5d | 10d | 20d | 30d |
| 3410 | O-H stretching | Cellulose | -2.31 | 6.14 | 16.89 | 22.48 |
| 2917 | C-H stretching | Cellulose, hemicellulose | -6.75 | -2.47 | 8.57 | 17.10 |
| 1650 | C=C stretching | Lignin | -6.66 | -5.16 | -7.12 | -1.92 |
| 1600 | Aromatic C=C stretching, C=O stretching | Lignin | 15.96 | 11.72 | 20.00 | 24.64 |
| 1372 | C-H stretching | Lignin | 10.50 | 21.14 | 23.35 | 31.98 |
| 1325 | C-O lilac ring | Lignin | 10.47 | 20.28 | 22.87 | 30.91 |
| 1258 | C-O alkyl ether stretching vibration | Lignin-hemicellulose ester bond | 6.04 | 9.09 | 10.25 | 16.35 |
| 1161 | C-O-C asymmetric stretching | Cellulose, hemicellulose | 3.80 | 22.52 | 20.10 | 22.72 |
| 898 | β(1,4) glycosidic bond | Cellulose amorphous | 20.06 | 11.71 | 26.89 | 23.72 |

| Table S5 Assignment of the peaks to functional groups, biomass components and their percentage changes relative due to pretreatments by FTIR in TW. | | | | | | |
| --- | --- | --- | --- | --- | --- | --- |
| Wave number (cm^-1^) | Asignment | Biomass component | Intensity (% relative percentage changes) | | | |
|  |  |  | 5d | 10d | 20d | 30d |
| 3415 | O-H stretching | Cellulose | -7.84 | -26.23 | -6.26 | -20.99 |
| 2920 | C-H stretching | Cellulose, hemicellulose | -18.00 | -22.97 | -31.28 | -35.03 |
| 1730 | C=O acetyl group | Hemicellulose | -15.41 | -6.75 | -20.42 | -21.29 |
| 1460 | Aromatic C=C stretching, C=O stretching | Lignin | -13.14 | -8.09 | -21.47 | -18.26 |
| 1425 | C-H out-of-plane deformation | Lignin | -7.99 | -4.17 | -16.50 | -14.98 |
| 1372 | C-H stretching | Lignin | -5.18 | -0.88 | -13.65 | -10.07 |
| 1327 | C-O lilac ring | Lignin | -5.51 | -2.51 | -13.48 | -5.91 |
| 1251 | C-O alkyl ether stretching vibration. | Lignin-hemicellulose ester bond | -8.96 | -8.66 | -17.65 | -12.04 |
| 1160 | C-O-C asymmetric stretching | Cellulose, hemicellulose | 3.80 | 22.52 | 20.10 | 22.72 |
| 1047 | C-O telescopic vibration | Cellulose, hemicellulose | 3.87 | -11.18 | 5.59 | 9.19 |
| 898 | β(1,4) glycosidic bond | Cellulose amorphous | 0.73 | 5.39 | 0.73 | 10.57 |

| 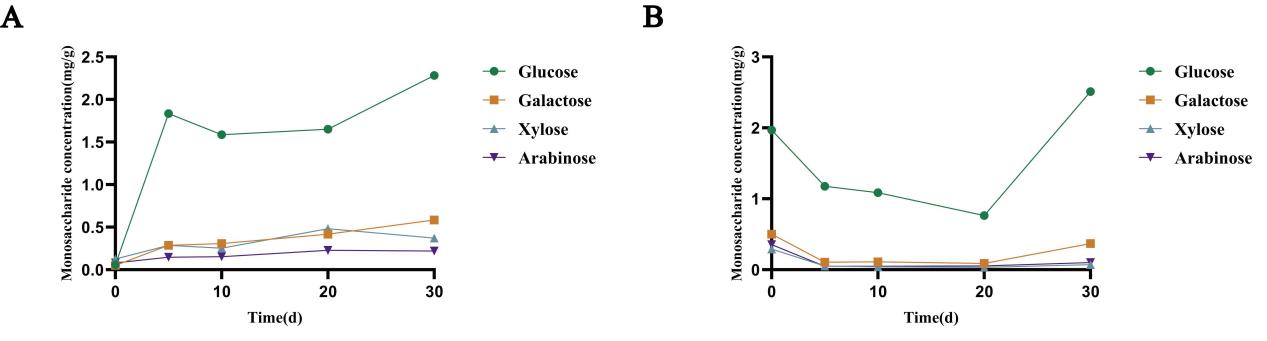 |
| --- |
| Figure S1 The content of four monosaccharides at different times in SSF. (A) TN group. (B) TW group. |
